# An assessment of extended pembrolizumab dosing in advanced non-small-cell lung cancer in the COVID-19 pandemic

**DOI:** 10.2217/imt-2022-0257

**Published:** 2023-07-11

**Authors:** Gordon Taylor Moffat, Lilian Hanna, Wilma Hopman, Andrea S Fung, Pierre-Olivier Gaudreau

**Affiliations:** ^1^Division of Medical Oncology & Hematology, Princess Margaret Cancer Centre, Toronto, ON, M5G 1X6, Canada; ^2^Department of Oncology, University of Toronto, Toronto, ON, M5G 1X6, Canada; ^3^Department of Medical Oncology & Hematology, Sunnybrook Health Sciences Centre, Toronto, ON, M4N 3M5, Canada; ^4^Department of Public Health Sciences, Queen’s University, Kingston, ON, K7L 3N6, Canada; ^5^Department of Oncology, Queen’s University, Kingston, ON, K7L 5P9, Canada; ^6^Canadian Cancer Trials Group, Cancer Research Institute, Queen’s University, Kingston, ON, K7L 2V5, Canada

**Keywords:** alternative dosing, immunotherapy, non-small-cell lung cancer, outcomes, pembrolizumab, safety

## Abstract

**Background:** There are limited clinical data comparing extended dosing (ED) versus standard dosing (SD) of pembrolizumab for metastatic non-small-cell lung cancer. **Methods:** This retrospective study included patients with metastatic non-small-cell lung cancer and PD-L1 tumor proportion score ≥50% treated with one or more cycles of single-agent pembrolizumab with SD or ED from January 2018 to December 2020. **Results:** A higher proportion of patients were alive in the ED group (vs SD) at 6 months (94 vs 51%), 12 months (94 vs 33%) and data cutoff (94 vs 26%) (p < 0.001 for all). The rate (44 vs 32%; p = 0.407) and severity of grade ≥3 immune-related adverse events were similar (50 vs 52%); however, ED patients more frequently discontinued treatment due to toxicity (45 vs 15%; p < 0.001). **Conclusion:** A greater proportion of ED patients were alive at data cutoff, and the rate and severity of immune-related adverse events were similar between groups.

## Plain language summary

Cancer in the lungs can be treated with drugs that use your immune system to kill cancer. This study showed that patients lived longer when the drugs were given further apart, and that the extended treatment was equally safe as the standard dosing schedule.

## Tweetable abstract

More patients were alive at data cutoff with extended dosing of pembrolizumab in patients with advanced #NSCLC and PD-L1 TPS ≥50%. However, a greater proportion of ED patients discontinued treatment due to toxicity. #Immunotherapy

Since 2019, the SARS-CoV-2 has led to a COVID-19 pandemic. Individuals with cancer are more susceptible to infections because of their immunocompromised state from cancer and anticancer treatments, in addition to their overall poor health status and chronic medical comorbidities [1–5]. In a multicenter study by Dai *et al.*, patients with cancer and COVID-19 had a higher observed death rate, admission rate to the intensive care unit, requirement of invasive mechanical ventilation and complication rate, and deteriorated more rapidly compared with COVID-19 patients without cancer [1]. Among those infected, lung cancer was the most frequent cancer type, and patients with metastatic disease had a higher risk than patients with nonmetastatic cancer [1].

Single-agent anti-PD-1 immune checkpoint blockade (ICB) has become the standard of care in treatment of Canadian patients with metastatic non-small-cell lung cancer (mNSCLC) and PD-L1 tumor proportion score (TPS) expression ≥50% based on overall survival (OS) benefits and manageable toxicity profile [6,7]. Clinical trials have previously evaluated both weight-based and fixed intermittent dosing of pembrolizumab [8,9]. Population pharmacokinetic analysis has shown that fixed dosing and weight-based dosing provides similar control of pharmacokinetic variability, with considerable overlap in the distributions of exposures, supporting suitability of a fixed dose of pembrolizumab 200 mg every 3 weeks [10]. OS benefits have also been confirmed using fixed dosing [7,9]. Consequently, pembrolizumab is currently administered every 3 weeks for up to 2 years in mNSCLC; however, the ongoing COVID-19 pandemic has changed everyday medical oncology practice significantly [11]. To minimize cancer patients’ risk of exposure to the novel virus, Cancer Care Ontario produced guidelines suggesting extended intermittent dosing schedules of ICB for NSCLC. The ‘Interim Cancer Drug Funding Measures for the Novel Coronavirus (COVID-19)’ published by Cancer Care Ontario stated that *“extended treatment breaks of publicly funded drugs may be required based on clinical discretion”* [12]. Specifically, the administration of pembrolizumab 4 mg/kg intravenously (up to a maximum of 400 mg) every 6 weeks was suggested as an alternative dosing schedule.

However, there is a paucity of formal literature evaluating extended intermittent dosing (ED) of ICB. Before the approval of fixed dosing, the first clinical trials involving pembrolizumab in NSCLC explored doses of either 2 or 10 mg/kg every 3 weeks or 10 mg/kg every 2 weeks [8,12]. Results showed that there was no difference according to dose or schedule [8,13,14]. Pharmacokinetic modeling has predicted that intermittent dosing of pembrolizumab (400 mg every 6 weeks) would produce similar efficacy and safety as compared with the currently approved regimen (i.e., 2 mg/kg up to 200 mg every 3 weeks) [15,16]. Evidence of extended intermittent scheduling efficacy and safety has been predicted through pharmacokinetic modeling, but formal real-world assessment of response and toxicity for such regimens is lacking in the NSCLC population [17]. Our study aimed to evaluate the real-world outcomes and safety profile of ED of single-agent pembrolizumab in patients with mNSCLC and PD-L1 TPS ≥50% during the COVID-19 pandemic compared with patients treated with the pre-COVID-19 standard dosing (SD) regimen.

## Patients & methods

### Study population

This retrospective study included all patients with histologically or cytologically confirmed mNSCLC and PD-L1 TPS ≥50% who received at least one dose of single-agent pembrolizumab in the first-line therapy setting at the Cancer Centre of Southeastern Ontario (ON, Canada) from January 2018 to December 2020. Patients with stage III disease (American Joint Committee on Cancer tumor node metastasis staging, 8th edition) were included if they were not candidates for surgical resection or definitive chemoradiation. Ethics approval from the institution was obtained before study commencement, and data were collected from a review of clinical, pathological and radiological reports. Baseline patient, tumor and treatment characteristics including age, sex, Eastern Cooperative Oncology Group (ECOG) performance status, tobacco smoking history, medical comorbidities, baseline autoimmune conditions, concomitant medications, date of pathological diagnosis, histological subtype, *EGFR*/*ALK*/*KRAS* mutation testing (using Oncomine Comprehensive Assay v. 3 next-generation sequencing), PD-L1 TPS (using the 22C3 IHC pharmDx assay), 8th edition American Joint Committee on Cancer tumor node metastasis staging at diagnosis, dates of first and last dose of pembrolizumab, number of treatment cycles received, date of the switch to alternative dosing pembrolizumab if applicable, the type and grade of immune-related adverse events (irAEs; as per Common Terminology Criteria for Adverse Events v. 5.0) if they occurred and reasons for treatment discontinuation were collected. All histological subtypes were included and classified as squamous cell carcinoma, non-squamous cell carcinoma, or other.

### Treatment

All patients included in the study initially received pembrolizumab at the SD of 2 mg/kg intravenous up to 200 mg every 3 weeks. If patients were switched to the ED group of 4 mg/kg intravenous (up to a maximum of 400 mg) every 6 weeks, the date of the switch and the first treatment on ED were recorded. The last treatment was recorded for both SD and ED patients, along with the number of cycles of treatment received on either dosing schedule.

### Study outcomes

The primary outcomes were the proportion of patients alive at 6 and 12 months and data cutoff, and the incidence of irAEs. Secondary outcomes included the median number of treatment cycles, time to toxicity, the type and grade of irAEs, reasons for treatment discontinuation, and OS (defined as time from treatment initiation to death from any cause). irAEs were documented based on type, grade, date of initial presentation, date of resolution, whether systemic therapy was continued or withheld and whether treatment was restarted if withheld. Subsequent episodes of irAEs were documented with the same categories of information.

### Statistical analysis

All statistical analyses were conducted using SPSS v. 25 (IBM Corp., NY, USA). Descriptive statistics were used to summarize clinical characteristics. Univariate analyses were completed using Pearson’s χ-square test or Fisher’s exact test to compare the categorical baseline patient, tumor and treatment characteristics of the two groups. Student’s *t*-tests, followed by Mann–Whitney *U* tests, were used for continuous data analysis. A Kaplan–Meier curve was used to compare time to death (or last seen) for the two groups. The designated threshold of statistical significance was p < 0.05, and no adjustment was made for multiple comparisons. The numbers of treatment cycles received by patients in each group were counted and represented as mean and median values. The number, rate and grade of irAEs were reported as percentages.

## Results

### Patient characteristics

A total of 90 patients were included in this study; baseline patient characteristics are summarized in Table 1. 60 patients (66.7%) were female; all patients had stage IV disease and most had nonsquamous histology (60/90; 66.7%) and baseline ECOG of 1 (82/90; 91.1%). All 90 patients had a history of smoking, with 65 former smokers (72.2%) and 25 current smokers (27.8%). Seven patients had a history of an autoimmune condition (7.8%), and the majority were in the SD group (6/7; 85.7%). The autoimmune conditions included rheumatoid arthritis (2/7; 28.5%), Crohn’s disease (1/7; 14.3%), atopic dermatitis (1/7; 14.3%), psoriasis (1/7; 14.3%), chronic urticaria (1/7; 14.3%) and polymyalgia rheumatica (1/7; 14.3%). Four patients had a baseline diagnosis of hypothyroidism; however, it is unknown whether this was due to an autoimmune etiology. None of the patients had a baseline use of a prednisone (or equivalent) dose of ≥10 mg within 30 days of initiation of therapy, but one patient was on a chronic dose of oral prednisone 5 mg daily for rheumatoid arthritis.

**Table 1.** Baseline clinicopathological and treatment characteristics of non-small-cell lung cancer patients receiving standard and extended pembrolizumab dosing during the COVID-19 pandemic.

	Standard dosing group (N = 72): n (%)	Extended dosing group (N = 18): n (%)	p-value
**Sex** Male Female	19 (26) 53 (74)	11 (61) 7 (39)	0.01^†^
**ECOG** 0 1 2 and above	4 (6) 64 (88) 4 (6)	0 (0) 18 (100) 0 (0)	0.56
**Smoking status** Former Current	52 (72) 20 (28)	13 (72) 5 (28)	1.00
**Stage** IV	72 (100)	18 (100)	NA
**Baseline bone metastasis** Presence Absence	21 (29) 51 (71)	5 (28) 13 (72)	0.91
**Baseline brain metastasis** Presence Absence	18 (25) 54 (75)	2 (11) 16 (89)	0.34
**Baseline adrenal metastasis** Presence Absence	8 (11) 64 (89)	3 (17) 15 (83)	0.69
**Baseline liver metastasis** Presence Absence	9 (12.5) 63 (87.5)	1 (6) 17 (94)	0.68
**Histology type** Squamous Nonsquamous Other^‡^	19 (26) 48 (67) 5 (7)	6 (33) 12 (67) 0 (0)	0.52
**Reason for treatment discontinuation** Immunotoxicity Progression Completed treatment Medical condition Death	10 (14) 26 (36) 2 (2.5) 5 (7) 22 (30.5)	5 (28) 1 (5) 5 (28) 0 (0) 0 (0)	<0.01^†^
**Still on treatment**	7 (10)	7 (39)	<0.01^†^
**Number of treatment cycles, median**	3.5	19^§^	<0.01^†^
**Alive at 6 months** (n = 54; 60%)	37 (51)	17 (94)	<0.001^†^
**Alive at 12 months** (n = 41; 46%)	24 (33)	17 (94)	<0.001^†^
**Alive at data cutoff** (n = 36; 40%)	19 (26)	17 (94)	<0.001^†^

† Statistically significant.

‡ Other histologies included poorly differentiated carcinoma with or without spindle cells.

§ On average, 11.5 cycles received on 3-weekly dosing before switch to 6-weekly dosing.

ECOG: Eastern Cooperative Oncology Group; NA: Not applicable.

The most common site of metastasis was bone (26/90; 28.8%), followed by brain (20/90; 22.2%). There was an even distribution of bone metastases between both groups of patients, but more patients with brain metastases in the SD group (Table 1). All patients had a PD-L1 TPS of ≥50%, 29 patients had a *KRAS* mutation (32.2%), and none had an *EGFR* or *ALK* mutation.

On χ-square analysis, there was no significant difference between the two groups based on smoking status, ECOG status, histology subtype, number of irAEs, or treatment withheld due to irAEs.

### Comparison of SD versus ED: effect on patient outcomes

**Figure 1.**
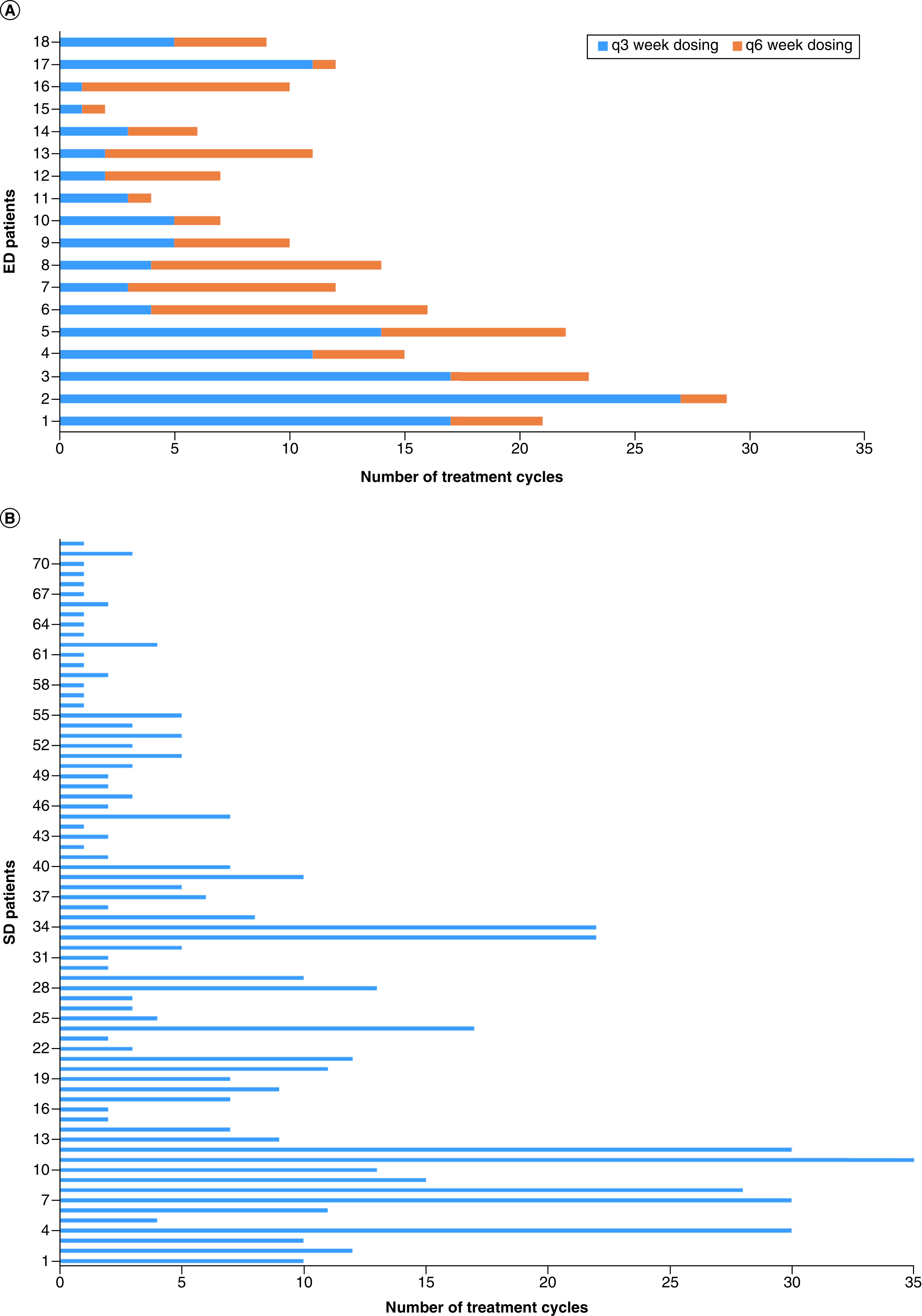
Number of treatment cycles per treatment group. **(A)** Number of treatment cycles received by NSCLC patients in the the extended dosing of pembrolizumab group during the COVID-19 pandemic group. **(B)** Number of treatment cycles received by NSCLC patients in the standard dosing of pembrolizumab group during the COVID-19 pandemic. ED: Extended dosing; NSCLC: Non-small-cell lung cancer; SD: Standard dosing.

Eighteen patients (20.0%) switched from SD to ED (Figure 1A). Overall, the ED group received more cycles of therapy (median = 19, with a median of 11.5 cycles received before the switch) compared with the SD group (median = 3.5) (p < 0.01; Figure 1A & B).

**Figure 2.**
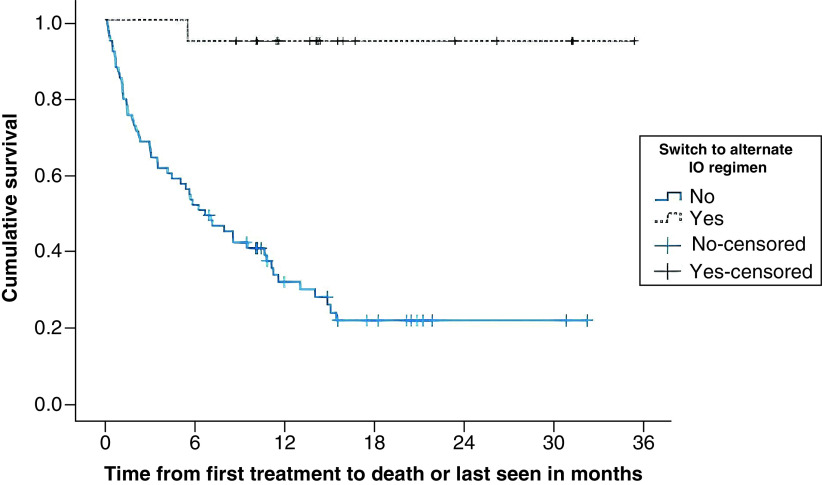
Overall survival: Kaplan–Meier curve for extended versus standard dosing of pembrolizumab in patients with non-small-cell lung cancer during the COVID-19 pandemic. IO: Immuno-oncology (i.e., pembrolizumab).

At 6 months, 54 patients (60%) were still alive, which dropped to 41 (46%) by 12 months and 36 (40%) at data cutoff. The proportion of patients alive was significantly higher in the ED versus the SD group (94% at all three time points vs 51, 33 and 26% at 6 months, 12 months and data cutoff, respectively; p < 0.001 for all; Table 1), and more patients were still on treatment in the ED group (39 vs 10%; p < 0.01) at the time of data cutoff. The median OS of the overall group was 10.6 months. A Kaplan–Meier analysis was significant for prolonged median OS of patients in the ED group (undefined) compared with the SD group (6.2 months) (p < 0.001; Figure 2).

Of the 90 patients evaluated in the total population, seven completed treatment (7.8%), 14 were still on treatment (15.6%), and 69 patients discontinued treatment (76.6%). In the ED group, the most common reasons for discontinuation of treatment were the completion of treatment (5/11; 45%) and immunotoxicity (5/11; 45%). In the SD group, the most common reason for treatment discontinuation was progression of disease (26/65; 40%), followed by death (22/65; 34%) and immunotoxicity (10/65; 15%).

### Immune-related adverse events

The rates of irAEs in the ED and SD groups were 44 and 32%, respectively (p = 0.41), and the median time to toxicity was 130 and 105 days, respectively (p = 0.44; Table 2). In total, irAEs occurred in 31 patients (34%): eight patients (26%) in the ED group and 23 patients (74%) in the SD group (Table 2). The majority of irAEs occurred within 3 months (n = 12; 39%) or between 3 and 6 months (n = 10; 32%) of starting immunotherapy, followed by between 6 and 12 months (n = 6; 19%) and >12 months (n = 3; 10%). The time between initiation of pembrolizumab and the occurrence of an irAE did not differ significantly between the two groups (p = 0.34; Table 2). No irAEs occurred after discontinuation or completion of therapy. In the ED group, three of the eight patients (37.5%) experienced an irAE before the switch to 6-weekly dosing, and five patients (62.5%) experienced irAEs after the switch. Of the three patients in the ED group who experienced an irAE before the switch in dosing schedule, one patient had their treatment withheld (33.3%) but then restarted. Of the five patients in the ED group who experienced an irAE after the switch in dosing schedule, four had their treatment withheld (80%) and none had their treatment restarted. The remaining patient continued with treatment but developed a second irAE (colitis), at which point treatment was discontinued. In the SD group, 17 patients had their treatment withheld (74%) due to irAEs, and five restarted treatment (29%). Overall, patients in the ED group were less likely to restart treatment if it was withheld (p = 0.02), and a higher proportion of ED patients discontinued treatment due to immunotoxicity compared with SD patients (45 vs 15%; p < 0.001). Within the ED group, the irAEs leading to treatment discontinuation included grade 2 pneumonitis, grade 3 colitis, grade 4 bullous pemphigoid and grade 4 fatigue.

**Table 2.** Characteristics of immune-related adverse events in non-small-cell lung cancer patients receiving standard and extended pembrolizumab dosing during the COVID-19 pandemic.

	Standard dosing group (N = 72): n (%)	Extended dosing group (N = 18): n (%)	p-value
**Rate of irAEs** (n = 31; 34%)	23 (32)	8 (44)	0.41
**Time between initiation of IO and irAE** <3 months 3–6 months 6–12 months >12 months	10 (44) 7 (30) 3 (13) 3 (13)	2 (25) 3 (38) 3 (38) 0 (0)	0.34
**Timing of irAE** During SD During ED	23 (100) –	3 (37) 5 (63)	<0.001^†^
**Median time to toxicity (quartiles)**	105 days (44–193)	130 days (75–212)	0.44
**Type of irAE** Thyroid dysfunction Pneumonitis Colitis Dermatitis Arthritis Fatigue Thrombocytopenia Vasculitis Adrenal insufficiency Myelitis Nephritis	4 (19) 7 (30) 4 (19) 1 (4) 1 (4) 1 (4) 1 (4) 1 (4) 1 (4) 1 (4) 1 (4)	1 (12.5) 1 (12.5) 2 (25) 2 (25) 1 (12.5) 1 (12.5)	–
**Grade of irAE** 1 2 ≥3	9 (39) 2 (9) 12 (52)	1 (12) 3 (38) 4 (50)	0.11
**Treatment withheld due to irAE** No (n = 10, 32%) Yes (n = 21, 68%)	6 (26) 17 (74)	4 (50)^‡^ 4 (50)^§^	0.38
**If withheld, treatment restarted** No (n = 16, 76%) Yes (n = 5, 24%)	12 (71) 5 (29)	4 (100) 0 (0)	0.02^†^

† Statistically significant.

‡ Three of these patients had irAEs during the 3-weekly dosing schedule.

§ All four patients had irAEs during the 6-weekly dosing schedule.

ED: Extended dosing; IO: Immuno-oncology (i.e., pembrolizumab); irAE: Immune-related adverse event; SD: Standard dosing.

The types of irAEs were similar between the groups and included thyroid dysfunction, pneumonitis, colitis, dermatitis and arthritis. The SD group had a more extensive range of irAEs, which included myelitis, nephritis, vasculitis and thrombocytopenia (Table 2). The rates of grade 3 or higher irAEs were similar between groups (ED: 50 vs SD: 52%). In the SD group, one patient with immune-related thrombocytopenia received intravenous immunoglobulin, and one patient with immune-related pneumonitis received intravenous immunoglobulin and mycophenolate mofetil, in addition to corticosteroids. Both patients died related to irAEs. There were no deaths related to irAEs in the ED group.

## Discussion

The KEYNOTE-010 and KEYNOTE-024 trials evaluated weight-based and fixed dosing of pembrolizumab, respectively, in the management of patients with mNSCLC and PD-L1 TPS expression [8,9]. Current evidence suggests that steady-state concentrations of pembrolizumab are achieved after 19 weeks, and maximum serum target engagement can be reached with trough levels of doses ≥1 mg/kg every 3 weeks [18,19]. Predicated on this knowledge, Lala *et al.* conducted a translational population pharmacokinetic modeling study that simulated pembrolizumab concentration time in 2993 patients from five clinical trials to examine the efficacy of a 6-weekly dosing schedule compared with the standard 3-weekly dosing schedule. The results demonstrated similar predictive exposures, drug concentrations (area under the curve) and safety profiles between the two dosing schedules [16]. Most of the data supporting the use of alternative dosing intervals of pembrolizumab rely on pharmacokinetic studies based on exposure–response relationships and through target engagement across various tumor sites [13]. Due to the COVID-19 pandemic, changes in clinical practice guidelines allowed the incorporation of 6-weekly dosing regimens into clinical practice in an attempt to limit patient visits and reduce the risk of exposure to COVID-19 for vulnerable patients with established increased mortality risk of infection [1,11,20–22]. These changes in clinical practice provided the opportunity to study the real-world outcomes and safety profiles of mNSCLC patients treated with SD versus ED of pembrolizumab, which added to the scarce information and provided insight into the controversy regarding optimal dosage and schedule [23–25].

The results of this retrospective study highlight that more patients treated with ED of pembrolizumab were alive at 6 months, 12 months and at the time of data cutoff than those treated with SD. Furthermore, we demonstrated that patients in the ED group received a greater number of treatment cycles than the SD group, which could translate to better-than-expected outcomes and survival. The ED group included a lower prevalence of brain metastases as compared with the SD group, which may have impacted the findings. In addition, a selection bias within the ED group cannot be excluded, as these patients were initially on the SD schedule and likely switched to the ED schedule given ongoing response to pembrolizumab and favorable tolerance until that point. In a retrospective analysis by Hijmering-Kapelle *et al.* that included both stage III and stage IV NSCLC patients (n = 205), the progression-free survival (PFS) and OS were comparable between ED and SD of palliative-intent pembrolizumab monotherapy or adjuvant durvalumab, whereas survival outcomes were improved in the ED cohort treated with palliative-intent nivolumab monotherapy [24]. Results with palliative-intent nivolumab provide support to our findings. However, similar to our study, the ED patients (n = 117) were initially treated with a SD schedule and subsequently switched to ED, and therefore a potential selection bias for survival cannot be excluded. Both studies contain potential confounding factors such as tolerability and clinical benefit that might influence whether patients were switched to the ED group or remained on the SD, the most important being if any immunotoxicity occurred. Additional confounding factors in the ED group of the Hijmering-Kapelle *et al.* study included fewer patients with ECOG >2, identified driver mutations and PD-L1 TPS of 0 [24].

ED strategies provide flexibility and convenience to patients, caregivers and hospitals, with the potential to improve patient-reported outcomes and quality of life and to reduce hospital and infusion-related costs [26–28]. The interim analysis of the KEYNOTE-555 trial (NCT03665597), a phase I open-label trial exploring stage III and IV melanoma patients treated with pembrolizumab 400 mg every 6 weeks, reported an overall response rate of 50.5% (95% CI: 30.4–60.6) compared with 35.1% in historical controls from KEYNOTE-001, KEYNOTE-006 and KEYNOTE-252 [29–32]. At a median duration of treatment of 8.2 months, 12.9% of patients had a complete response, and 39.6% of patients had a partial response, compared with 14 and 28%, respectively, in the KEYNOTE-006 trial with SD treatment [31]. However, the KEYNOTE-006 trial’s median follow-up was 57.7 months [31]. For KEYNOTE-555, the median PFS was 13.8 months (95% CI: 3.0–not reached) and the estimated PFS rates were 56.5 and 54.3% at 6 and 12 months, respectively, which is improved from previously reported studies [29].

In a pooled analysis of NSCLC patients treated with pembrolizumab in the KEYNOTE-001 and KEYNOTE-010 clinical trials, the incidence of irAEs was expected to be similar between the ED and SD groups [8,16,26,30]. In our study, the rate, type and grade of irAEs were similar between the two treatment groups, and comparable with those reported in a 2020 real-world cohort study of 1010 patients with NSCLC treated with pembrolizumab (32.9%), along with other published studies using SD [33–36]. In another small cohort published by Abdelsalam *et al.*, of 21 lung cancer patients treated with single-agent pembrolizumab, patients who were switched to ED experienced at least one AE, with the majority being fatigue and skin toxicity (pruritus) [23]. In comparison, the most common irAEs in our ED cohort were skin-related AEs and colitis.

In our study, the time to toxicity was longer in the ED group as compared with the SD group (130 vs 105 days), but both were longer than the previously reported median onset of 40 days in real-world analyses [36,37]. Interestingly, the majority of the irAEs in the ED group occurred after the switch from SD, and immunotoxicity was found to be one of the major reasons for discontinuation of therapy (unlike the SD group) and clinically meaningful. Although the discontinuation rate is likely the result of high-grade irAEs in the ED group, the basis for the specific timing of irAE occurrence following the switch remains uncertain. Although previous pharmacokinetic models had predicted a similar toxicity profile between ED and SD, patients in our cohort were already on SD at the time when the dose was increased as part of the ED regimen. Given that the half-life of pembrolizumab is 22 days, it is possible that the total effective dose may have been higher than 400 mg when the first ED treatment was administered. In the interim analysis of KEYNOTE-555 (NCT03665597), a phase I open-label trial exploring stage III and IV melanoma patients treated with pembrolizumab, the drug concentrations at 400 mg 6-weekly dosing were slightly higher in the first 21 days and slightly lower in the last 21 days compared with the 200 mg and 2 mg/kg 3-weekly dosing [38]. The serum concentrations after the first 400 mg infusion rapidly approached steady-state concentration levels and were maintained for the remaining treatment duration. The average concentration of pembrolizumab with 400 mg 6-weekly dosing was predicted to be similar to that with the 200 mg 3-weekly dosing but 35% higher than with the 2 mg/kg 3-weekly dosing. Further, the maximum steady-state peak plasma concentration with pembrolizumab 400 mg 6-weekly dosing was 59% higher than with 200 mg 3-weekly dosing and 113% higher than with 2 mg/kg 3-weekly doing. The analysis also reported that among patients who switched from pembrolizumab 200 mg every 3 weeks to 400 mg every 6 weeks, the pharmacokinetic parameters at steady state were similar to those observed with continuous 400 mg 6-weekly dosing [39]. While this study used weight-based dosing, the overall irAEs were similar between the weight-based dosing in KEYNOTE 011 and the fixed dosing used in KEYNOTE 024 and 042, which in theory could overtreat patients who weigh <100 kg. Conversely, those patients who weigh >100 kg could be overdosed based on weight-based dosing calculations [9,30,40].

It is unlikely that polypharmacy and drug metabolism would affect the effective dose of pembrolizumab, as previously reported studies have demonstrated no significant difference in the incidence of grade 3–5 irAEs between older or younger patient cohorts [41,42]. In a real-world, multicenter, retrospective analysis of patients receiving pembrolizumab for any indication, Rowe *et al.* showed a trend toward a higher incidence of any-grade irAEs in the ED group, albeit not statistically significant [43]. In support of our findings, patients were more likely to develop an irAE after switching from 3- to 6-weekly dosing, especially if no irAE occurred during the 3-weekly schedule [43]. In the aforementioned study by Hijmering-Kapelle *et al.*, there was also a higher rate of irAEs in the ED pembrolizumab monotherapy cohort as compared with the SD group (p = 0.02); however, the increase in irAEs did not result in an increased number of grade ≥3 irAEs between the ED and SD groups, or events leading to treatment interruption or discontinuation [24]. Of note, the rate of discontinuation due to toxicity was lower compared with our study. By contrast, in a study by Mac *et al.* of stage III NSCLC patients on adjuvant durvalumab, the rates of irAEs were not significantly different between the ED and SD groups, but trended higher in the SD group. The observed rate could be related to the stage of disease or the drug used [25].

Several studies have explored the predictive cost savings of weight-based, fixed and pharmacokinetic-derived dosing [26,44,45]. In a study by Goldstein *et al.*, personalized body-weight dosing of pembrolizumab was equally efficacious as fixed dosing but more cost-efficient, with an estimated annual saving of $0.825 billion a year in the USA [44]. In a study at the University of Washington, weight-based dosing of pembrolizumab reduced spending by 19% [46]. This strategy has already been adopted in Canada, but the availability of appropriate vial sizes creates logistical challenges [11,47]. Later, in 2020, Goldstein *et al.* reported that a weight-based approach of pembrolizumab 4 mg/m^2^ every 6 weeks was not only effective but beneficial during the COVID-19 pandemic to reduce patients’ risk of exposure to infection, and resulted in financial savings to the healthcare system [11]. Further, in an exploratory population pharmacokinetics study of nivolumab, ED predicted a potential cost saving of 70% and was a practical strategy during the COVID-19 pandemic [26,48]. However, the financial impact of utilizing ED strategies is not fully understood.

Our study has a few limitations. First, the results must be interpreted with caution given the small number of patients included in the ED group for analysis. On the other hand, recently published cohorts yield support for our findings. Secondly, there is a substantial issue of immortal time bias, in that those who switched to ED had obviously not died or progressed on SD, and had also received substantially more cycles of SD, which suggests that they would have had a better prognosis. For this reason, mortality outcomes were provided at 6 months and 12 months after initiation of treatment, in addition to the study end. Similarly, the rate of irAEs would also be affected by the length of time on treatment, as those who had a longer time on treatment would presumably have more time to develop irAEs. Thirdly, patients who develop an irAE are more likely responding to immunotherapy and trend toward having a long survival [49,50]. Fourthly, our study was unable to evaluate patients who received ED of pembrolizumab from the initiation of ICB therapy; all patients in this group were started on the standard-of-care SD schedule and then switched to an ED schedule. As there are few real-world and clinical data on starting patients on ED, we suspect many providers initiated an SD regimen to assess tolerance before considering ED. Future studies including patients who started on ED may reduce the possible bias found in our study. Fifthly, this was a single-institution study, and its mainly Caucasian patient population may not entirely reflect mNSCLC patients with other ethnicities being treated with ICB. However, our cancer center serves a catchment area of over 600,000 people and is located at a tertiary academic hospital. Lastly, a possible limitation is the common practice of telemedicine during the COVID-19 pandemic, which may have had implications during patient encounters and for the physician’s decision to switch the patient to an ED schedule, assess for early signs of irAEs and manage other clinical concerns, all of which may affect patient outcomes or the continuation of treatment. Other studies operating during the COVID-19 pandemic may have experienced similar limitations.

## Conclusion

In this retrospective analysis, the real-world outcomes and safety of ED of pembrolizumab in the management of NSCLC were examined due to the incorporation of ED regimens into clinical practice during the COVID-19 pandemic to reduce vulnerable cancer patients’ risk of exposure to infection by requiring them to make fewer visits to the cancer center. A greater proportion of ED patients were alive at the time of data cutoff, but this may reflect an immortal time bias and a selection bias, as patients were more likely switched considering disease stability. The rate and severity of irAEs were similar between the ED and SD groups, and irAEs in this study were consistent with those reported in previous trials with SD scheduling. However, a high proportion of irAEs in the ED group occurred following the switch, in many cases justifying treatment cessation. As published data comparing ED and SD schedules of ICB in NSCLC remain sparse and confounding factors limit the interpretation of results, a possible impact of ED on survival and toxicity should be further validated in prospective cohorts in both advanced and curative disease settings, as well as studies exploring the use of combination immunotherapy and chemotherapy, and those being treated with flat doses and other non-pembrolizumab doses if ED dosing is adopted in the future.

**Summary points**

- Of the 90 non-small-cell lung cancer patients evaluated, 18 (20%) switched to extended dosing (ED). The proportion alive at data cutoff was significantly higher in the ED versus the standard dosing (SD) group (94 vs 26%; p < 0.001) and the ED group received more cycles of therapy (median: 19 vs 3.5; p < 0.01).
- The median time to toxicity was 130 (ED) versus 105 (SD) days, and the rate (44 vs 32%; p = 0.407) and severity of grade ≥3 immune-related adverse events (irAEs) were similar (50 vs 52%).
- A greater proportion of ED patients discontinued treatment due to toxicity (45 vs 15%; p < 0.001); five of eight irAEs (62.5%) in the ED group occurred after switching, resulting in treatment discontinuation.
- The types of irAEs were similar between the groups and included thyroid dysfunction, pneumonitis, colitis, dermatitis and arthritis.
- The majority of irAEs occurred within 3 months (n = 12; 39%) or between 3 and 6 months (n = 10; 32%) of starting immunotherapy, followed by between 6 and 12 months (n = 6; 19%) and >12 months (n = 3; 10%). The time between initiation of pembrolizumab and irAE did not differ significantly between the two groups (p = 0.34). No irAEs occurred after discontinuation or completion of therapy.
- The impact of ED on survival and toxicity should be further validated in prospective cohorts.
